# Combined oncolytic virotherapy gold nanoparticles as synergistic immunotherapy agent in breast cancer control

**DOI:** 10.1038/s41598-023-42299-4

**Published:** 2023-10-06

**Authors:** Majid S. Jabir, Ahmed M. Al-Shammari, Zainab O. Ali, Salim Albukhaty, Ghassan M. Sulaiman, Sabrean F. Jawad, Sawsan S. Hamzah, Asad Syed, Abdallah M. Elgorban, Rajalakshmanan Eswaramoorthy, Nouf S. S. Zaghloul, Ali G. Al-Dulimi, Mazin A. A. Najm

**Affiliations:** 1 Division of Biotechnology, Department of Applied Sciences, University of Technology, Baghdad, 10066 Iraq; 2 Experimental Therapy Department, Iraqi Center for Cancer and Medical Genetics Research, Mustansiriyah University, Baghdad, Iraq; 3 Department of Chemistry, College of Science, University of Misan, Maysan, 62001 Iraq; 4 College of Medicine, University of Warith Al-Anbiyaa, Karbala, Iraq; 5 Department of Pharmacy, Al-Mustaqbal University College, Babylon, Iraq; 6 College of Dentistry, Department of Basic Sciences, Ibn Sina University of Medical and Pharmaceutical Sciences, Baghdad, Iraq; 7 Department of Botany and Microbiology, College of Science, King Saud University, P.O. 2455, 11451 Riyadh, Saudi Arabia; 8 Department of Prosthodontics, Saveetha Dental College and Hospitals, Saveetha Institute of Medical and Technical Sciences (SIMATS), Chennai, 600 077 India; 9 Bristol Centre for Functional Nanomaterials, HH Wills Physics Laboratory, Tyndall Avenue, University of Bristol, Bristol, BS8 1FD UK; 10 Department of Dentistry, Bilad Alrafidain University College, Diyala, 32001 Iraq; 11 Pharmaceutical Chemistry Department, College of Pharmacy, Al-Ayen University, Thi-Qar, Iraq; 12 Center of Excellence in Biotechnology Research, King Saud University, Riyadh, Saudi Arabia

**Keywords:** Biotechnology, Cancer

## Abstract

Combining viruses and nanoparticles may be a way to successfully treat cancer and minimize adverse effects. The current work aimed to evaluate the efficacy of a specific combination of gold nanoparticles (GNPs) and Newcastle disease virus (NDV) to enhance the antitumor effect of breast cancer in both in vitro and in vivo models. Two human breast cancer cell lines (MCF-7 and AMJ-13) and a normal epithelial cell line (HBL-100) were used and treated with NDV and/or GNPs. The MTT assay was used to study the anticancer potentials of NDV and GNP. The colony formation assay and apoptosis markers were used to confirm the killing mechanisms of NDV and GNP against breast cancer cell lines. p53 and caspase-9 expression tested by the qRT-PCR technique. Our results showed that combination therapy had a significant killing effect against breast cancer cells. The findings demonstrated that NDV and GNPs induced apoptosis in cancer cells by activating caspase-9, the p53 protein, and other proteins related to apoptosis, which holds promise as a combination therapy for breast cancer.

## Introduction

According to the World Health Organization, breast cancer BC affects approximately one in eight women during their lifetime in Europe and North America^1^. Along with standard treatment modalities of surgery, radiotherapy, chemotherapy, immunotherapy, targeted therapy, and hormone therapy, new approaches such as gene therapy, nanotherapy, and viral therapy have also been tried to treat cancer^2–4^. When rituximab and Newcastle Disease virus (NDV) were combined, cytotoxicity that was p53-independent was promoted. It is a cutting-edge treatment modality for some hematologic malignancies^5^. However, nanotechnology is a particularly promising area for new medical applications. Nanotechnology is the study of materials with sizes between 1 and 100 nm. These developments have had a profound impact on many industries, including medicine, pharmaceuticals, biology, and chemistry. Therefore, the term nanobiotechnology has been proposed. Nanoparticles have many biological applications such as biosensing, antimicrobial, molecular imaging, and anticancer therapeutic compounds^6–8^. Nanotechnology is the term used to describe various arrangements of matter at the nanoscale. Numerous related domains in physics, chemistry, engineering, and biology have developed as a result of this technique. Recently, nanotechnology has attracted great attention, especially in the field of medicine (also known as nanomedicine)^9^. Nanoparticles (NPs) are used for imaging, therapy, and diagnostics of cancer-related diseases. Different types of NPs with different capabilities, such as drug delivery, have been developed to enhance already available drugs. Quantum dots and organic and inorganic nanoparticles (such as lipid-based and polymeric NPs) suggest that targeting solid tumors will result in successful oncotherapy^10, 11^. The distinguishing characteristics that differentiate this approach from conventional therapies include increased drug solubility, systemic stability, target delivery to tumors, control drug release, and lower toxicity. A significant accumulation of NPs is also present in tumors in comparison to normal tissues, accompanied by an increase in permeability and retention^12^. Combination strategies aim to kill tumor cells while preventing the establishment of resistance to treatment by employing a variety of mechanisms of action^13^. Selectivity between normal and malignant cells is necessary for effective anticancer treatments. As a result, the choice of an oncolytic virus (OV) based on the selective replication of oncolytic viruses in tumor cells with low toxicity to normal cells, tumor cells especially susceptible to oncolytic viruses, can be produced by flaws in the interferon pathway or oncogenic transformation^14^. Newcastle disease virus (NDV), a promising oncolytic virus with a single-stranded, negative-sense, non-segmented RNA genome, belongs to the genus Avulavirus in the family Paramyxoviridae^15^. NDV is classified into three pathotypes: lentogenic (avirulent), mesogenic (intermediate), and velogenic (virulent) depending on the cleavage site in the F protein, which is considered a major determinant of virulence^16^. NDV-mediated oncolysis includes multiple antitumor mechanisms, such as direct cytolysis secondary to viral replication^17, 18^ and caspase apoptosis induction and activation^19–21^. Various studies revealed that NDV could induce apoptosis by activating extrinsic (Death Receptor-mediated Apoptosis) and intrinsic mitochondrial apoptotic pathways^22^. Indirect mechanisms such as immune-mediated anticancer activity. NDV infection of tumor cells leads to the expression of viral glycoproteins (HN and F) on the surface of the tumor cell, which changes the surface adhesion of the tumor cell to erythrocytes and lymphocytes, leading to upregulation of T-cell activation^23–25^. NDV has been shown to have potent antiangiogenic effects by triggering an acute disruption of the tumor vasculature^26^ or inhibiting the release of angiogenic factor from cancer cells^27^. NDV has also been reported to induce inhibition of glycolysis and starvation of cancer cells^28^. Kadhim et al*.* demonstrated that TMZ-PLGA-NPs are a useful modality for the treatment of gliomas, through NDV mediation in the AMGM5 human glioblastoma cancer cell line^29^. Oncolytic virotherapy showed to be effective when combined with other anticancer agent and work in synergisim specialy with nanoparticles such as silver nanoparticles^30^. Its also noticed that oncolytic NDV Iraqi starin showed synergestic anticancer effect when combined with phytochemicals such as *Cyperus rotundus* L. alkaloid extracts against digestive system tumors^31^. Moreover, Oncolytic NDV AMHA1 starin showed to be selective and effective agianst cancer cells that can avoid killing normal cells in 3-D culture systems^32^. The safety and immunogenicity of viral vectors are challenged, given that they are produced from viruses that have previously been acquired by spontaneous infection. Although vector engineering advances and surface modifications have improved their safety profile, the problem remains a significant concern in this regard. Therefore, nanoparticles are being investigated as a potential alternative for current oncolytic viral delivery systems. In the present study, we evaluated the use of gold nanoparticles with their distinctive properties to increase the sensitivity of breast cancer cells to oncolytic NDV, to enhance apoptosis induction, as well as being selectively toxic in cancer cells, and to induce apoptosis by activating caspase-9, the p53 protein, and various other proteins involved in apoptosis.

## Materials and methods

### Gold nanoparticles (GNPs)

GNPs were obtained from Sigma-USA and the specification revealed that nanoparticles were spherical in shape and size range of 10–15 nm*.* GNPs were characterized using SEM, and TEM techniques as shown in Supplementary Fig. 1.

### Cell culture

Human breast cancer cell lines Michigan Cancer Foundation (MCF-7), Iraqi human breast cancer cell line (Ahmed Murtadha Jabria 13; AMJ13), normal epithelial cells (HBL-100), murine mammary adenocarcinoma tumors (AN3), and monkey kidney epithelial cells (Vero Cell) were provided by the experimental therapy department, ICCMGR, University of Mustansiriyah, Baghdad, Iraq. In MEM medium, HBL-100 and MCF7 cell lines were cultured (US Biological, USA). AMJ13 cells, on the other hand, were grown in RPMI-1640 medium (US Biological, USA), which was supplemented with penicillin–streptomycin and 10% fetal bovine serum (FBS) (Capricorn-Scientific, Germany). The cells were then incubated at 37 °C in a humidified atmosphere containing 5% CO_2_.

### NDV propagation

A frozen seed − 20 C of the AMHA1 Iraqi strain of Newcastle disease virus was provided from ICCMGR, University of Mustansiriyah, Baghdad, Iraq. The stock was propagated in the embryonated chicken egg supplied by the Al-Khalil hatchery in Iraq. The stock was then removed from the allantoic fluid and the debris was cleaned by cold centrifugation (3000 rpm, 30 min at 4 °C). NDV titers were computed using a 50% tissue culture infective dose on the Vero cell line.

### Cytotoxicity assay

In a 96-well microplate, MCF-7, AMJ13, and HBL-100 cells were cultured at a density of 1 × 10^4^ per well and left to grow overnight or for 24 h at 37 °C until an inverted microscope showed a confluent monolayer. The MTT assay was used to measure cytotoxicity. The concentrations of diluted GNPs were (10 µg/ml, 20 µg/ml, and 30 µg/ml), and NDV in diluted multiplicities of infection (MOI) of 1, 3, 5, and 10 were successfully achieved. After 72 h, the medium was discarded and each well received 50 µl of MTT dye solution at a concentration of 2 mg/ml (Bio-World, USA). This was left to sit for 2 h and then 50 µl of dimethylsulfoxide (DMSO) was added to dissolve the dye (Santa Cruz Biotechnology, USA). 15 min were spent incubating the plates. The optical density of both cells (treated and untreated) was evaluated with a 492 nm ELISA plate reader^33, 34^.

1 $$\mathrm{Cytotoxicity \% }= (\mathrm{OD \,control}-\mathrm{ OD\, treated}) /\mathrm{OD \,control }\times 100,$$

where the (OD control) is the mean optical density for untreated cells in 3 plates, while the (OD treated) is the mean optical density for treated cells in 3 plates^35^.

### Combined cytotoxicity assays and Chou–Talalay analysis

The doses in this experiment were selected on the basis of the previous cytotoxicity MTT assay. We took the concentration around the IC_50_ value for both NDV and GNPs and combined them. AMJ13, MCF7, and normal cells were seeded at a density of 1 × 10^4^ cells/well into 96-well plates and incubated for 12 h. NDV was first added to different MOIs and incubated for 2 h, followed by the addition of GNPs at the selected concentration for the measurement of growth inhibition, in which each MOI of virus mixed with these GNPs to finally have 12 combinations groups. As described earlier, growth inhibition was measured after 72 h of incubation by the MTT assay. NDV and GNPs were studied in non-constant ratios to determine synergism using the Chou–Talalay combination index (CI) calculated using CompuSyn software (CompuSyn Inc., Paramus, NJ, USA) to analyze the combined effect of NDV and GNPs. CI values of 0.9–1, < 0.9, and > 1.1 indicate additive, synergism, and antagonism, respectively.

### AO/EtBr and DAPI staining

The apoptotic rates of treated and untreated breast cancer cells, as well as normal cells, were measured using dual AO/EtBr staining^36, 37^. In a 48-well plate containing 1 × 10^4^ cells/well, the cells were seeded for 24 h before treatment with NDV and GNPs alone. Then, they were mixed by adding NDV first and then GNP in an incubator at 37 °C for 72 h before stained with AO/EtBr. After incubation, cells were washed in triplicate with PBS and then stained for 30 s with a solution containing 50 μl of stain mixture and 10 μl each of AO and EtBr, and 1 ml of PBS. Finally, the dye was discarded and visualized with a fluorescent microscope^38^. DAPI staining was also used as an apoptotic confirmation assay.

### Measurement of reactive oxygen species (ROS) production in MCF-7 and AMJ-13 cells

According to the manufacturer’s instructions, the GENMED intracellular ROS red fluorescence determination kit (GENMED, Shanghai, China) was used for visualized ROS production in MCF-7 and AMJ-13 cells^39^.

### Flow cytometry

This assay was performed according to manufactured protocols for detecting apoptosis using Annexin V and for intracellular ROS production using the DCFH-DA probe.

### Real-time PCR

The expression levels of p53 and caspase-9 mRNA in treated breast cancer cells were measured using QPCR. Based on the standard sequences of the primer sets, we got from the National Center for Biotechnology Information (NCBI) database. cDNA was made by treating cells with DNase and then treating them with Superscript II reverse transcriptase (Invitrogen, Cat. No. 18,064-071, USA). The RNeasy Mini kit from Qiagen (Cat. No. 74,104, UK) was used to remove the total RNA from the cells. The preparation of each reaction mixture for qRT-PCR included 7.5 μL of SYBR green, 0.3 μL of ROX, 0.3 μL of related primers, and 1μL of cDNA. When 5.6 μL of distilled water was added, the final volume of 15 μL was reached. The Fast SYBR Green master mix (Applied Biosystems, UK) was mixed with the 7900HT fast system (Applied Biosystems). The mean relative values of the genes were calculated using established methods after normalizing the gene expression levels to the TATA-binding protein-encoding gene (Livak and Schmittgen).

### Measurement of apoptosis-related proteins for NDV-GNPs treatment

The expression response of the apoptosis-related protein to NDV, GNPs and combination was measured using the RayBio® C-Series Apoptosis Antibody Array 1 kit (RayBiotech Life, Inc., USA). MCF-7 cells were seeded in four tissue culture flasks (10^6^ cells per flask) and incubated at 37 °C with 5% CO_2_. After 24 h, the medium was removed. Three tissue culture flasks were exposed to NDV, GNPs, and their combination for 24 h; the untreated flask was considered a control treated with serum-free media only. Cells were lysed using 1 ml of lysis buffer for each flask, pulled into Eppendorf tubes, and left for 5 min for protein extraction. Finally, the extracted proteins were calculated by nanodrop (Thermofisher, USA) and normalized. Antibody array against human apoptosis was incubated overnight with 250 µg of proteins extracted from treated and untreated cells. A Biospectrum AC ChemiHR device was used to scan the membranes that were used in the process of quantifying the apoptosis array data. ImageJ was used to perform the statistical analysis and the expression levels of signal folds of each sample were calculated using the manufacturer’s guidelines^40^.

### Animal tumor model

This study used a transplantable line of murine mammary adenocarcinoma tumors (AN3). It is derived from an albino Swiss mouse with a spontaneously arising mammary tumor. Inbred syngeneic female mice are used to maintain the AN3 tumor line. Swiss albino female mice (8–10) weeks old (15–25 g) weight were purchased and maintained according to the guidelines of the ICCMGR animal house. AN3 cells (10^6^/100 μl/site) into the right flanks of albino mice to investigate tumors. When the tumor nodules reached 200 mm in diameter, the experiment was started. Studies were carried out following the recommendations of the ARRIVE guidelines under the guidance of the Animal Care and Ethics Committee ( approved no. UOT-ASD-020919) in the Division of Biotechnology, Department of Applied Sciences, University of Technology, Baghdad, Iraq, and according to the U.S. National Institutes of Health (NIH) Guide for the Care and Use of Laboratory Animals (NIH Publication No. 86–23, revised in 1996). Therefore, all methods were performed in accordance with relevant guidelines and regulations.

### Experimental design

The animals were randomly divided into four groups of six animals each: The GNPs treated group was injected intratumorally with 1 mg/kg daily for four days, the Newcastle disease virus treated group was injected intratumorally, with 10 × 10^7^ infectious viral particles infused every 30 min until the full dose was administered. The GNPs-NDV combination-treated group was co-administered with both (GNPs at 1 mg/kg daily for four days, then one single dose of NDV at (1 × 10^7^), and mice in the control group were left untreated until the end of the experiment. After 30 days, the mice were sacrificed.

### Evaluation of antitumor efficacy

The diameters of the tumors (length and width) were measured three times a week using a caliper. Tumor volume was calculated using the following formula (0.5 × lengths × width × width) as mean ± SEM for each group. Tumor volume was normalized to tumor volume on day zero, which was the time when treatment was initiated. The following equation calculates tumor growth inhibition (TGI):

2 $$\mathrm{TGI\% }=\frac{\mathrm{ tumor\, volume\, in\, the\, untreated\, group }-\mathrm{ tumor\, volume \,in\, the\, treated\, group}}{\mathrm{ Tumor \,volume\, of \,untreated \,group }} \times 100 ,$$

### Statistical analysis

Data were analyzed using a Graph Pad prism. A one-way ANOVA one-way test was used to compare the groups and *p* < 0.05 was considered significant. Data are represented as mean ± S.D^41^.

## Results and discussion

### Antiproliferative activity of NDV and GNPs against breast cancer cells

**Figure 1:**
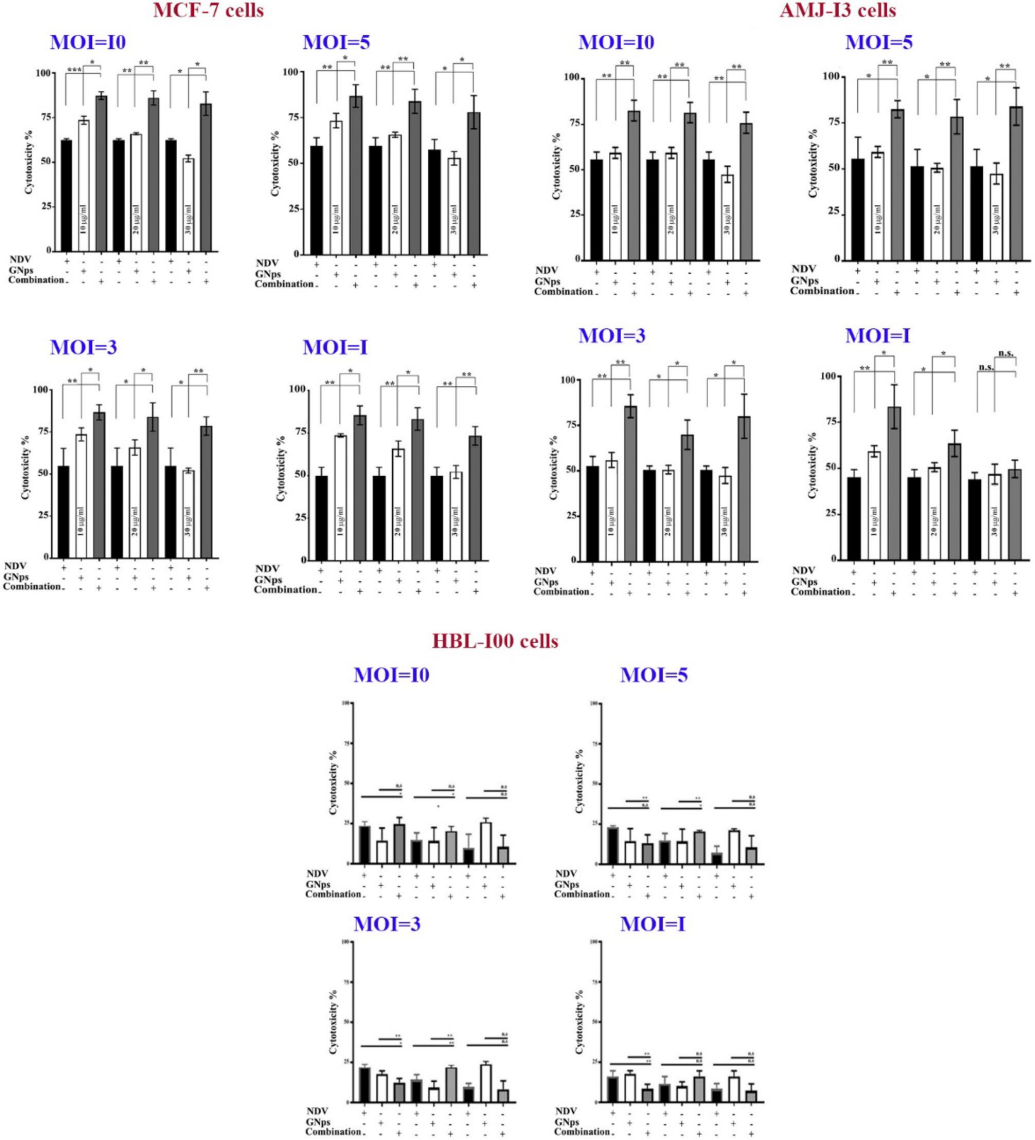
Cytotoxic activity of NDV and GNPs against MCF-7, AMJ-13, and HBL-100 cell lines. Untreated cells were used as control here for normalization. Data are represented as mean ± S.D. from three independent experiments. Asterisk (*) indicated a significant difference (p < 0.05). (**) indicated a significant difference (p < 0.01). While (***) indicated a significant difference (p < 0.001).

Different MOIs of NDV and different concentrations of GNPs were tested against breast cancer and normal cells using the MTT cytotoxicity assay (Fig. 1). The results showed the percentage of cytotoxic effect (CT%) in breast cancer cells, with no noticeable percentages in normal cells. The results demonstrated that the GNPs/NDV have a better anticancer efficiency than the GNPs or NDV alone. NDV has been shown to have potent antiangiogenic effects by triggering an acute disruption of tumor vasculature^26^ or inhibiting the release of angiogenic factors from cancer cells. NDV has also been reported to induce inhibition of glycolysis and starvation of cancer cells^27^. TMZ-PLGA-NPs are a useful modality for the treatment of gliomas, through NDV mediation in the AMGM5 human glioblastoma cancer cell line^29^. Additionally, an attenuated oncolytic strain of measles was found to work synergistically with silver nanoparticles to induce the killing of breast cancer cells^30^. Furthermore, the combination of oncolytic NDV with phytochemicals was also effective in inducing synergistic anticancer effects^31^. A study of Khoobchandani and his coworkers focused on the role of green nanotechnology in the treatment of breast cancer^42^. The study findings suggested that Nano Swarna Bhasma has the potential adjuvant therapeutic agent to reduce the detrimental effects of standard chemotherapy treatments. The use of green nanotechnology-compositions based on nano-Ayurvedic medicine, such as NSB medication, has been shown to have a low harmful effect on normal cells and tissues. As a result, nano-Ayurvedic treatment modalities present practical possibilities. Opportunities, as well as actions that are comprehensive and very effective in the care and treatment of patients suffering from cancer all over the world. Other study investigated the role of mangiferin-functionalized gold nanoparticles (MGF-AuNP) as an immunomodulatory agent and their application in the treatment of prostate cancer in vitro and in vivo models^43^. The outcome of the study demonstrated the role of MGF-AuNPs as an immunomodulatory action in prostate cancer by increasing the level of antitumor cytokines (IL-12 and TNF-α), and decreasing the levels of pro-tumor cytokines (IL-10 and IL-6). Furthermore, this study investigated the ability of MGF-AuNPs to promote splenic macrophages and their effect on the NF-kB signaling pathway.

### Combination cytotoxicity assays and Chou–Talalay analysis

**Figure 2:**
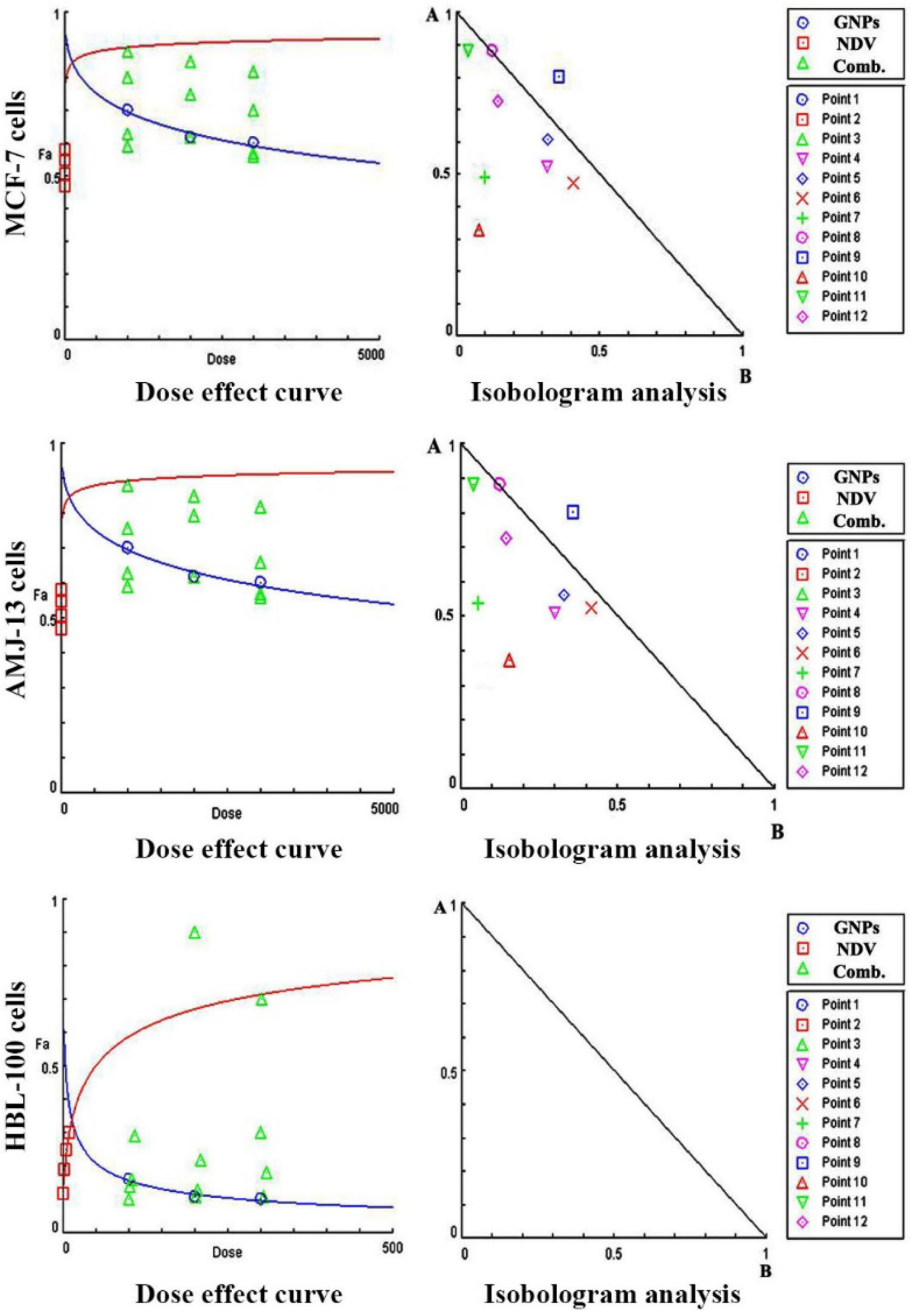
Synergistic effect of NDV and GNPs on breast cancer cell lines. Point 1 (10 µg/ml GNPs and MOI 10), Point 2 (20 µg/ml GNPs and MOI 10), Point 3 (30 µg/ml GNPs and MOI 10), Point 4 (10 µg/ml GNPs and MOI 5), Point 5 (20 µg/ml GNPs and MOI 5), Point 6 (30 µg/ml GNPs and MOI 5), Point 7 (10 µg/ml GNPs and MOI 3), Point 8 (20 µg/ml GNPs and MOI 3), Point 9 (30 µg/ml GNPs and MOI 3), Point 10 (10 µg/ml GNPs and MOI 1), Point 11 (20 µg/ml GNPs and MOI 1), Point 12 (30 µg/ml GNPs and MOI 1).

To evaluate the therapeutic efficacy of NDV and GNPs as a combination and their potential cytotoxicity, we examined the cytotoxicity ratio of the NDV (1, 3, 5, and 10 MOI) and GNPs (10 µg/ml, 20 µg/ml, and 30 µg/ml) alone and then each MOI of NDV with as concentration to get 12 combinations. The MTT cell viability assays were conducted in human breast cancer cell lines (AMJ13 and MCF-7) and normal cells (HBL-100). This study accredited the Chou-Talalay method for the study of drug combination, based on the (median-effect equation), which is derived from the principle of mass action law, the resulting combination index (CI) theorem of Chou-Talalay offers a quantitative definition of (synergism (CI < 1), antagonism (CI > 1) and additive effect (CI = 1), in treatment combinations. In addition, this theory presents algorithms for automated computer simulation of synergism and/or antagonism at any effect and dose level, as shown in the CI plot and isobologram, respectively. The combination index-isobologram equation method allows computerized quantification of synergistic, additive, and antagonism effects. Therefore, combination therapies have become a standard in several areas such as cancer, as described by^44^. To study the potential interaction between NDV and GNPs treatment in vitro, the effectiveness of the combined treatment of three concentrations of GNPs (10 µg/ml, 20 µg/ml, and 30 µg/ml) with NDV in MOI (10, 5, 3, and 1) was evaluated using cancer cell lines MCF-7, AMJ-13, AMN-3 and the normal cell line HBL as shown in (Fig. 2). Cells were treated with NDV and GNPs alone and with combination treatment (NDV and GNPs) and growth inhibition was determined after 72 h of incubation time by the MTT assay for synergistic effect using isobologram analysis. Furthermore, the results show that in all treatments with NDV and GNPs alone against MCF-7 cells at all MOI and NPs concentrations, the combination treatment revealed a higher growth inhibition percentage than NDV or GNPs alone. The results against normal cell lines show that all NDV and GNPs treatments alone and the combination doses on HBL cells at all concentrations of MOI and NPs numbers and the combination treatment has a weak effect on the normal cell line HBL compared to the effect on three cancer cell lines. CI was estimated from dose–effect data from single and combined treatments using the CompuSyn Isobologram. CI < 1 indicates synergism; CI = 1 to 1.1 indicates an additive effect, and CI > 1.1 indicates antagonism. CI in AMJ-13 was synergistic in eight combination points (CI < 1) with only one additive effect. In eight combination points, the CI against MCF-7 cancer cells was less than 1. Finally, in normal cells, the combination points were higher than one in all 12 combinations, indicating the antagonist effect, which is a neglected effect since there was no killing effect reaching 50% in all combinations tested.

### Apoptosis and morphological changes in breast cancer cells

**Figure 3:**
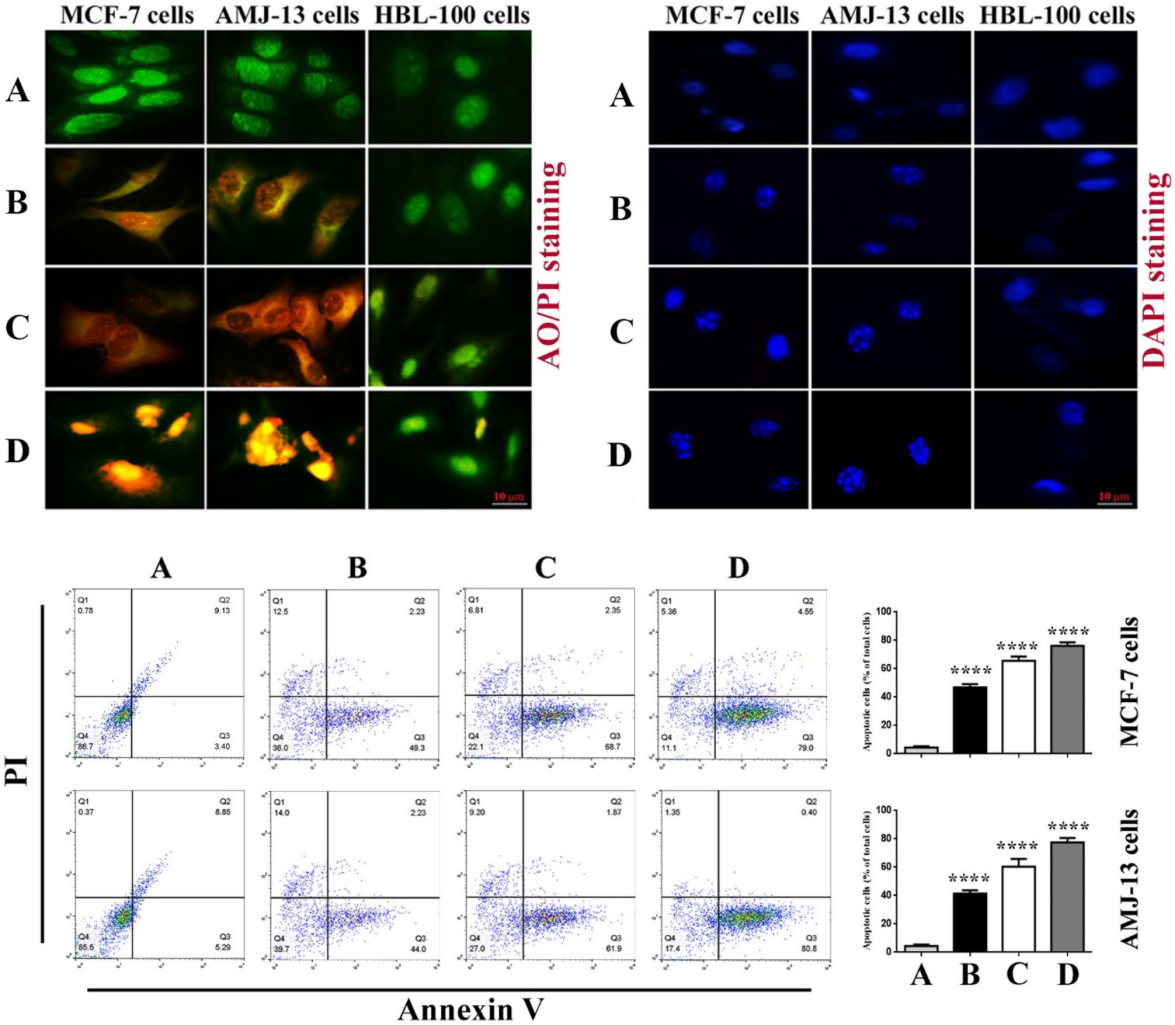
Apoptosis markers in breast cancer cells after being treated with NDV and GNPs. The upper left panel represents the results of the AO/PI staining assay. The upper right panel represents DAPI stain showing nucleus fragmentation in breast cancer cells. The lower panel represented the detection of apoptosis by flow cytometry in breast cancer cells after treatment with NDV and GNPs. Data are represented as mean ± S.D. from three independent experiments. The asterisks (****) indicated a significant difference (p < 0.0001). A untreated control cells. B NDV treated cells. C cells treated with GNPs. D NDV and GNPs treated cells.

**Figure 4:**
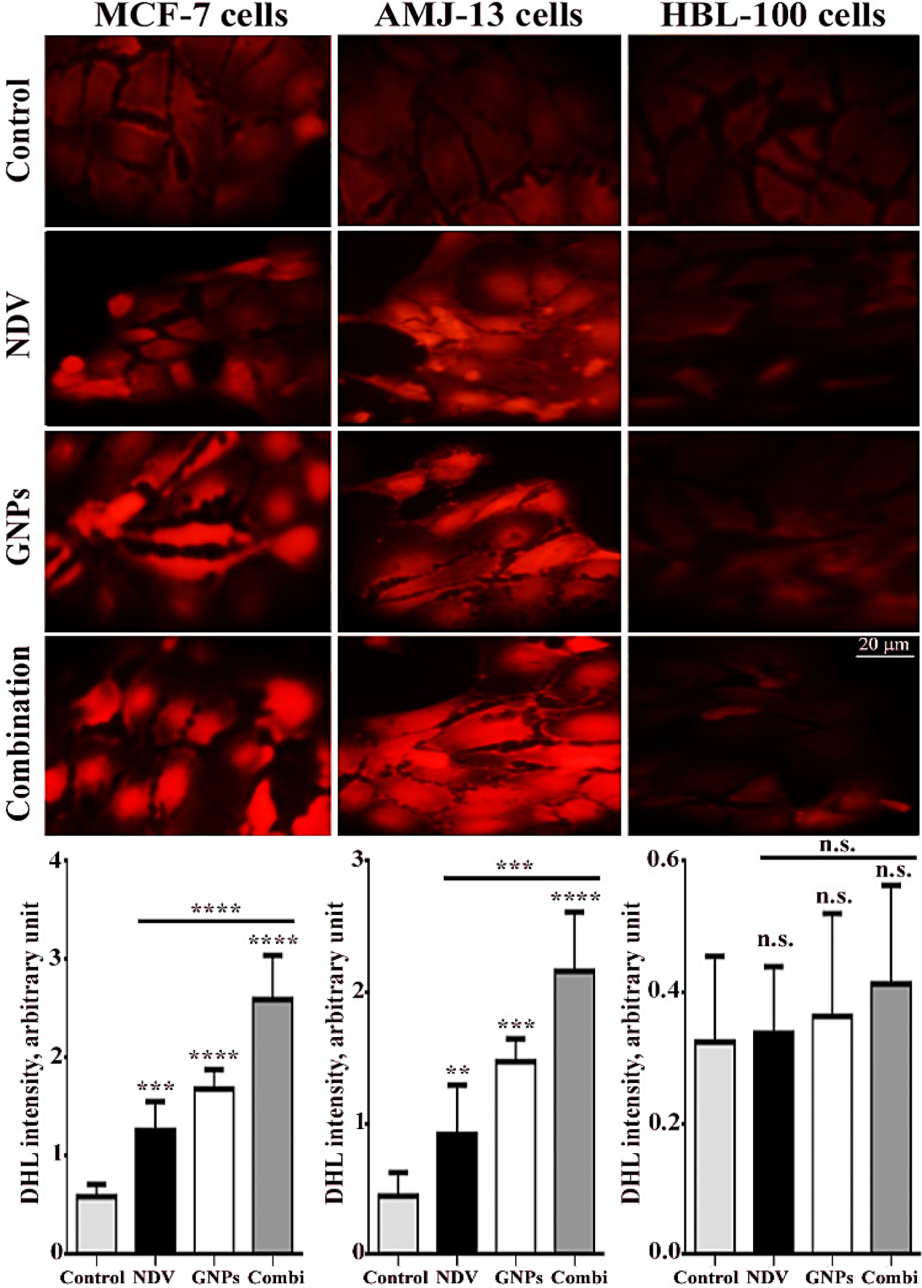
NDV and GNPs increase the production of ROS in breast cancer cells. Data are represented as mean ± S.D. from three independent experiments. The asterisks (**) indicated a significant difference (p < 0.01). (***) indicated a significant difference (p < 0.001). While (****) indicated a significant difference (p < 0.0001).

The ability of the combination of NDV and GNPs to induce apoptosis in treated breast cancer cells was confirmed by double acridine orange/ethidium propidium stain. As shown in Fig. 3(uppper left panel), apoptotic cells have been detected in both breast cancer cell lines (AMJ-13 and MCF-7), which were treated with NDV and GNPs alone and in combination, while viable green cells have been detected in the untreated and normal cell line that emitted green fluorescence. However, NDV-GNPs treated cancer cells emit red fluorescence significantly more than monotherapies. To confirm these results, the percentage of apoptotic cells by staining the cancer cells with annexin V-FITC using flow cytometry were determined. The flow cytometry findings showed that cells undergoing apoptosis were labeled with annexin V in quadrant Q3. Figure 4(lower panel) shows dot plots of breast cancer cell lines after being treated with NDV and GNPs for 24 h. In control MCF-7cells, the majority (86.7%) of the cells were viable and non-apoptotic, in MCF-7 NDV, GNPs-treated cells decreased in viable cells and increased in cells undergoing apoptosis 49.3%, 68.7%, and 79.0% respectively. The percentage of apoptotic cells in untreated AMJ13 was 5.29%. In AMJ13 cells treated with NDV and GNPs, the percentage increased to 44.0%, 61.9%, and 80.8%, respectively. An increasing percentage of dead or apoptotic cells was observed when breast cancer cells were treated with NDV and GNPs alone or combined. Furthermore, in Fig. 4(top right panel), as in NDV and GNPs treated breast cancer cells, nucleus fragmentation was observed in MCF-7 and AMJ-13 cells, while control cells possessed uniformly stained nuclei. In the current study, NDV and GNPs were used as a novel combination and compared their effect on monotherapies on in vitro human breast cancer cell lines. Naturally occurring oncolytic viruses emerge as novel tools for the selective growth and killing of various tumor cells^45^. Furthermore, one of the main antitumor activities is the induction of apoptosis, which can be caspase-dependent and independent^46, 47^. In comparison to NDV and GNPs alone, the combination of the two had the greatest ability to kill cancer cell lines. The combination had a more potent cytotoxic effect on the breast cancer cell line, while having a negligible effect on normal cells.

### ROS generation in MCF-7 and AMJ-13 cells

**Figure 5:**
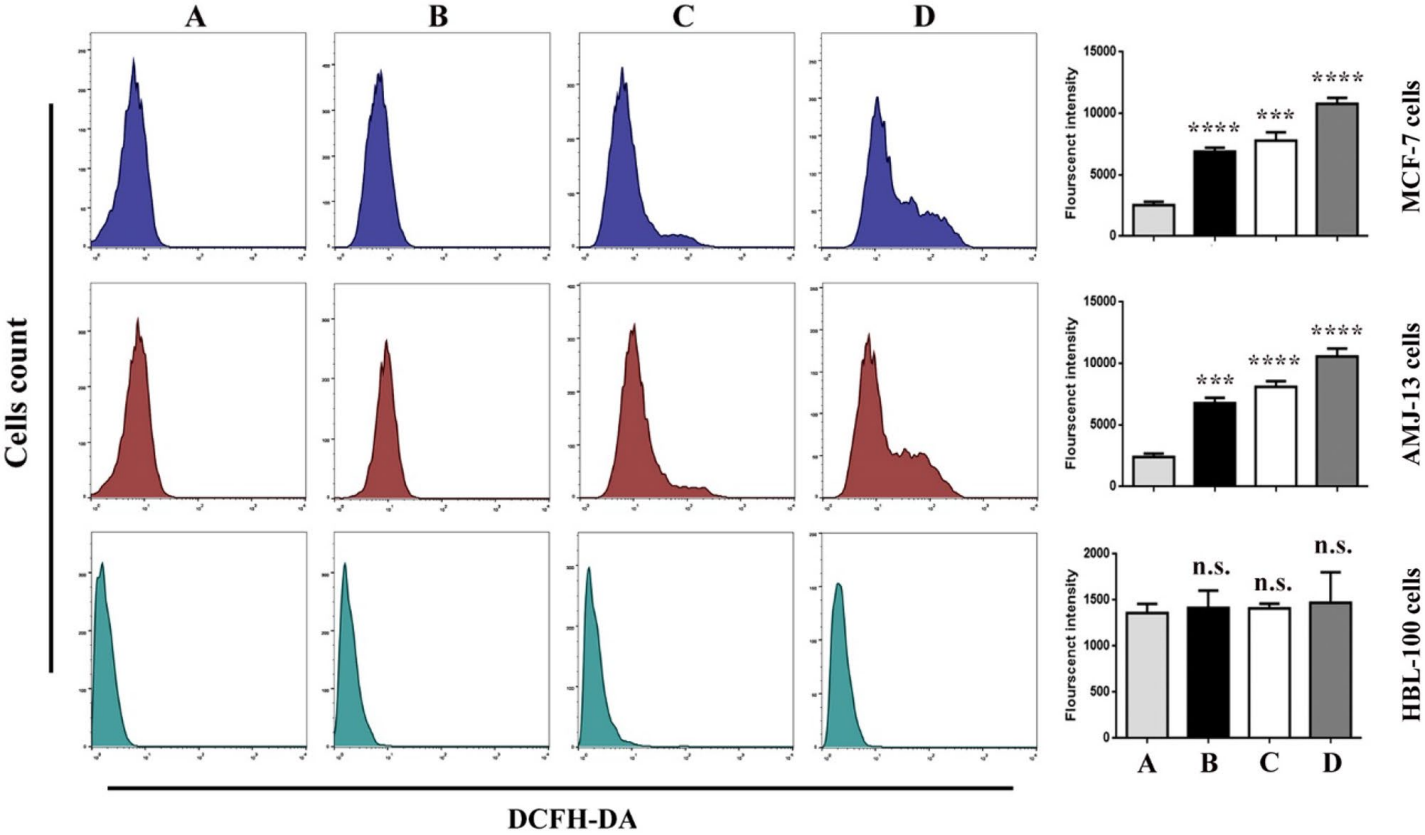
Intracellular ROS assayed with a DCFH-DA probe in breast cancer cell lines. (**A**) untreated control cells. (**B**) cells were treated with NDV. (**C**) cells were treated with GNPs. (**D**) cells were treated with NDV and GNPs. Data are represented as mean ± S.D. from three independent experiments. Asterisks (***) indicated a significant difference (p < 0.001). While (****) indicated a significant difference (p < 0.0001). (**A**) untreated control cells. (**B**) NDV treated cells. (**C**) cells treated with GNPs. D, NDV, and GNPs treated cells.

As a result, in the current study, ROS accumulation was examined in breast cancer cells after NDV and GNPs treatment. As illustrated by a representative dihydroethidium staining in Fig. 4, cells treated with NDV and GNPs showed up-regulation of ROS. Furthermore, combination therapy markedly increased ROS accumulation in breast cancer cell lines. The ROS were then assayed using a DCFH-DA probe as shown in Fig. 5. The results demonstrated that the ROS level increased when breast cancer cells were treated with NDV and GNPs.

### GNPs and NDV upregulated the expression of p^53^ and caspase-9

**Figure 6:**
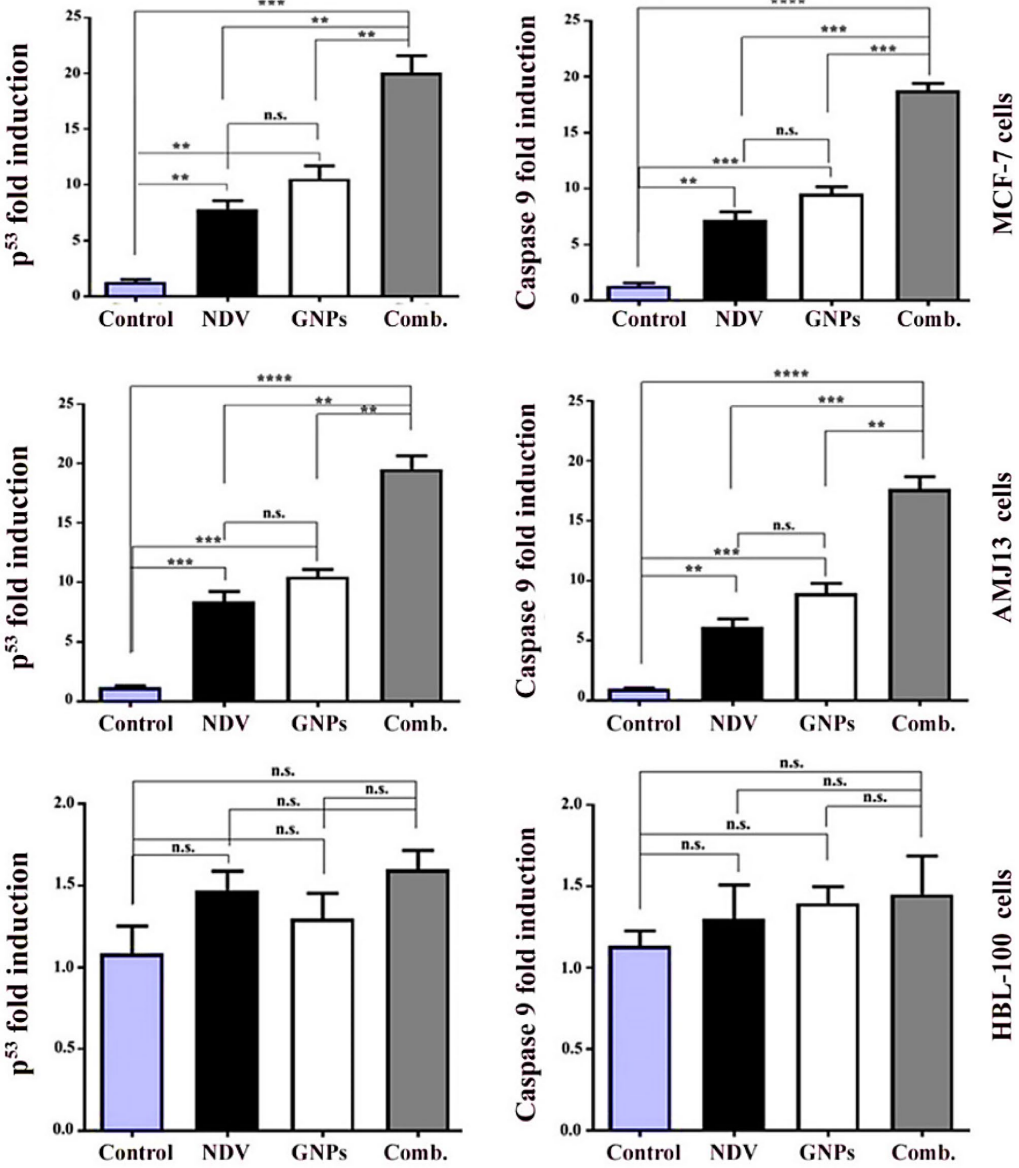
Upregulation of P^53^ and Caspase-9 in cancer cells after treatment with NDV and GNPs. Data are represented as mean ± S.D. from three independent experiments. The asterisks (**) indicated a significant difference (p < 0.01). (***) indicated a significant difference (p < 0.001). While (****) indicated a significant difference (p < 0.0001).

P^53^ and caspase-9 expression was evaluated in MCF-7 and AMJ-13 cell lines followed by treatment with GNPs and NDV, and the RT-PCR assay to assess apoptosis in treated and control groups. The PCR results demonstrated a significant difference in the expression of P^53^ and caspase-9 in the treated and untreated control group, as shown in Fig. 6. MCF-7 and AMJ-13 cells treated with GNPs and NDV expression of Caspase-9 and p53 expression were up-regulated. The two primary mechanisms necessary for the induction of apoptosis are the intrinsic pathway and the extrinsic pathway mediated by the death receptor (regulated at the level of mitochondria). The Fas-associated death domain (FADD) is recruited through molecule signals (Fas / FasL) on the surface in the extrinsic pathway^48^. The procaspase-8 gene can be activated by the FADD-Fas complex. Apoptosis is induced by the death-inducing signaling complex (DISC), a dynamic and important complex that can activate and induce caspase-3^49^. The actions of caspase-9 and p53 were investigated using real-time PCR. The findings showed that when MCF-7 and AMJ-13 cells were treated with GNPs and NDV, P53, as well as caspase-9, were significantly up-regulated compared to untreated cells.

### Apoptosis proteomic profile

**Figure 7:**
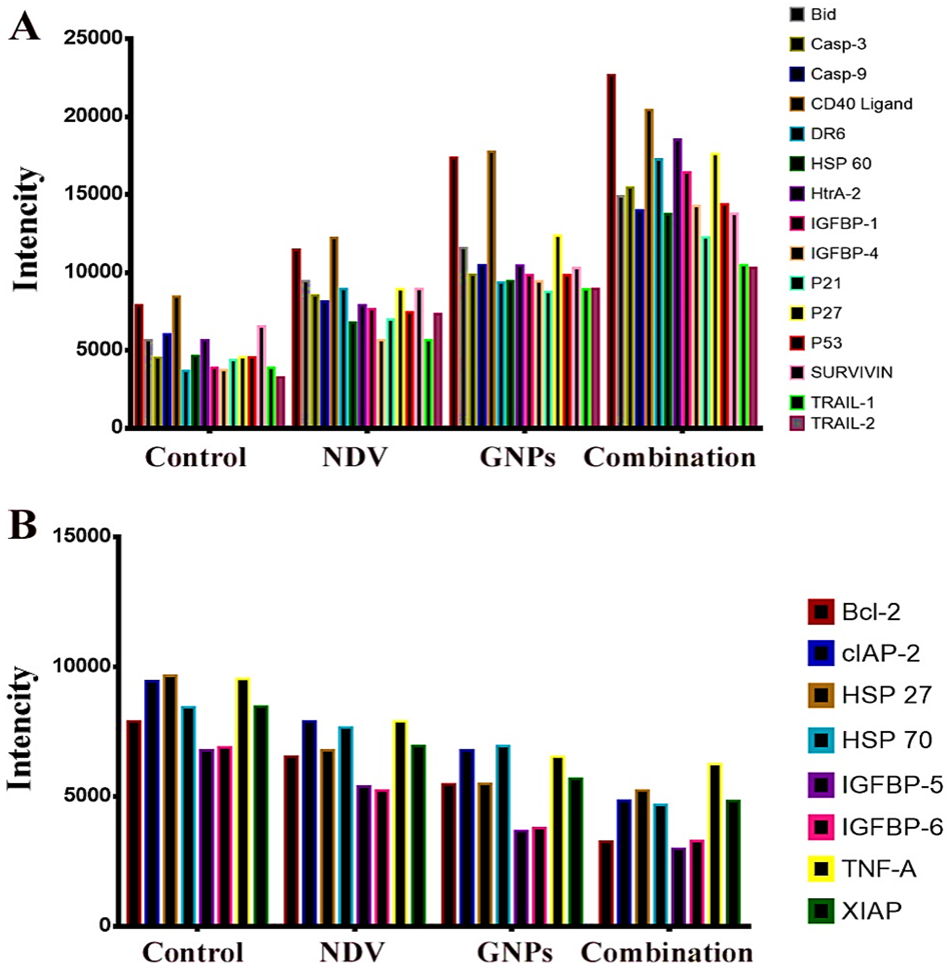
The combined therapy of NDV-GNPs up- and down-regulated apoptosis pathway proteins. (**A**) representing up-regulated apoptosis proteins. While (**B**) represents down-regulated apoptosis proteins.

**Figure 8:**
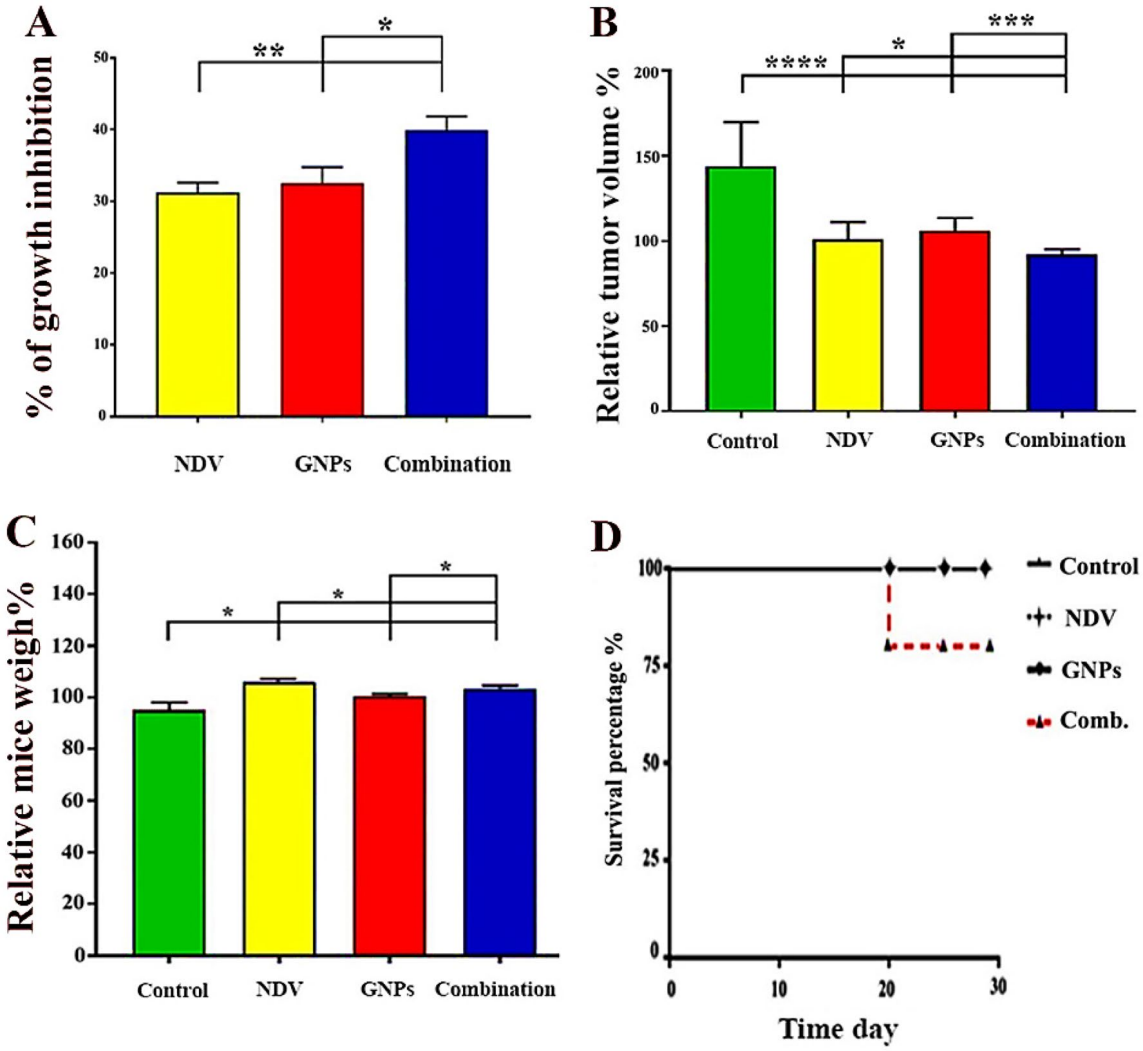
Effect of GNPs, NDV, and combination therapy for tumor model in albino Swiss mice. (**A**) represents % of growth inhibition. (**B**) represents the relative tumor volumes in response to GNPs, NDV, and combination therapy. (**C**) refer to relative mice weight %. (**D**) represents the survival curve of animals. The tumor reduction in the combination group is greater than NDV and GNPs alone. Data are represented as mean ± S.D. from three independent experiments. The asterisks (*) indicated a significant difference (p < 0.05). (**) indicated a significant difference (p < 0.01), (***) indicated a significant difference (p < 0.001). While (****) indicated a significant difference (p < 0.0001).

To investigate the mechanism of death induction by the combination of GNPs and NDV, an array of apoptosis proteins was used to study the expression of apoptosis proteins in the treated and untreated breast cancer cell line MCF-7 as shown in (Fig. 7A and B). We used the human apoptosis protein array to examine the main proteins involved in cell death and apoptosis after 24 h of treatment of MCF-7 cells with GNP and NDV. We noticed changes in these proteins, as shown in Fig. 7. Several proteins were either up- or down-regulated obviously, it depends on how they interacted with the pathway of apoptosis. Furthermore, several such proteins, including Bim, BAX, SMAC, Bad, cytochrome C, and HtrA-2, are essential components of the intrinsic apoptotic pathway; After 24 h with GNPs and NDV alone or in a mixture with MCF-7 cells, these proteins were significantly upregulated. Bid, caspase-3, CD40 ligand, DR6, survivin, Hsp60, HtrA-2, IGFBP-1, IGFBP-2, IGFBP4, p21, p27, p53, and TRAIL-1 and TRAIL-2 were present (Fig. 7A). On the other hand, the expression of Bcl-2, cIAP-2, Hsp27, Hsp70, IGFBP-5, IGFBP-6, TNF-A and XIAP was inhibited (Fig. 8B). Several investigations have shown that Bcl-2 proteins, including BAX, Bad, Bim, Bcl-2, and Bcl-w, are essential components of the mitochondrial pathway. These proteins transport cytochrome c from the mitochondria to the cytoplasm of the cell. This results in the generation of an apoptosome and the stimulation of caspase-9 downstream molecules, which facilitate the signaling of caspase-3 and caspase-7^49^. The finding of the investigation of the expression of cellular proteins indicates that the apoptotic process is endogenous. Once the markers SMAC/DIABLO and HtrA2/Omi are present, cIAP-2 and XIAP are less likely to be produced. The lower levels of protein expression demonstrate this. Furthermore, previous research has shown that the tumor suppressor protein p53 is essential to cause apoptosis. Additionally, p53 may interact with the mitochondrial pathway or stop the cell cycle by controlling the Bcl2 protein family or causing the expression of p21^50^. Furthermore, p53 is involved in the production of p27, which interacts with the Bax protein and causes apoptosis to occur more quickly^51^. During apoptosis, HSP70 and HSP27 were both downregulated, which was another finding of the current work. Furthermore, when MCF-7 cells were exposed to GNPs and NDV, protein levels such as CD40, Bad, DR6, Bid, caspase-3, caspase-8, TNF, Fas, and TRAIL-4 increased. Several clinical correlation studies have shown that breast cancer cell resistance is caused by the high level of anti-apoptotic Bcl-2 proteins and survivin. Several pieces of evidence suggest that most chemotherapeutic agents work against cancer by causing apoptosis; therefore, antiapoptosis may be one of the main reasons why they are less effective^52^. It was interesting to find that the same stress signals that cause apoptosis and make more apoptotic proteins also cause heat shock proteins (HSP) to be produced and released^53^. HSPs are molecular chaperones, which are protective proteins that are generally overproduced by cells when they are exposed to things that damage proteins, such as high temperatures, low oxygen levels, heavy metals, drugs such as papaverine, and viral infections NDV or in combination^54^. The present findings can show that both an important apoptotic protein and HSPs have gone up. However, there are many ways to explain how HSPs protect cells from apoptosis. HSPs can interact with cytochrome c to prevent its dimerization with Apaf-1, thus inhibiting the development of the apoptosome complex. HSPs can also prevent pro-apoptosis factors such as p53, Bax, and Bid from performing their functions^55^. Another remarkable finding is that reduction in the expression of insulin-like growth factor-1 (IGF-1 and 2) in vitro and in vivo has very important therapeutic value, as reported that the IGF-1-stimulated signal transduction pathway stimulates breast cancer cell proliferation, survival, and metastasis^56^. Present findings reported downregulation of cIAP2 after papaverine-NDV combined therapy in vitro and in vivo, which effectively boosts apoptosis by triggering caspase 3/7 in cancer cells. This makes cIAP2 inhibition of therapeutic value^57^. Increased expression of caspase-3 confirms the ICC finding of our experiments in vitro and in vivo. The results of this study’s apoptosis proteome array experiment showed that MCF-7 cells treated with GNPs and NDV can induce apoptosis through both the intrinsic (via mitochondria) and extrinsic (via Fas, FTNFR, and TRAIL) pathways.

### Antitumor effects of GNPs-NDV: in vivo model

An animal tumor model was used to test the antitumor activity of the GNPs–NDV combination in vivo. AN3 cancer cells were injected into Swiss albino mice to monitor tumor growth. Mice were randomly assigned to four groups when the tumors reached 0.5–1 cm in diameter, as described above. The relative tumor volumes were plotted over 30 days, with the first day before treatment being treated as 100%. There was a continuing increase in relative tumor size for the negative control group (without treatment) from the beginning to the end of the experiment; unlike the combination-treated group, the experiment ended with a clear decrease in tumor size in which there was a significant difference between the untreated control and the treated group. Furthermore, the combination therapy group induced the highest rate of inhibition of tumor growth at the end of the experiment, followed by the GNPs group, and finally, the NDV group had the lowest rate of inhibition of growth with significant differences in growth inhibition between combination therapy and monotherapy, as shown in Fig. 8. Figure 8D explains the survival prolog between treatment groups throughout the experiment, which shows that all mice in the four groups lived after the doses of the treatment for 30 days, except for one death that belongs to the fourth group on the nineteenth day after the last dose.

## Conclusions

The present study aimed to estimate the increase in the sensitivity of cancer cells to oncolytic virotherapy using GNPs, when combined with other antitumor agents, NDV is an attractive anticancer agent. NDV was synergistic with GNPs on breast cancer cell lines in vitro through the involvement of caspase-induction and its ability to induce a wide range of apoptotic proteins. The present findings indicated a novel combination that can enhance the antitumor property for both oncolytic virotherapy and GNPs. NDV and GNPs were more effective in inhibiting breast cancer cell growth than NDV and GNPs alone. To investigate the molecular mechanisms of apoptosis in response to NDV infection, GNPs, and their combination, the array of apoptosis proteins was used. Results of the microarray analysis in which NDV, GNPs, and combination can induce apoptotic factors within the first 24 h after exposure. This effect was greater in the combination treated groups, which may explain the greater cytotoxic effect in breast cancer cell lines. We found that combined therapy with NDV-GNPs induced increased expression of some pro-apoptotic proteins and decreased expression of antiapoptotic factors as a mechanism for combination therapy to induce cell death. According to the findings, cancer cells can be treated with NDV and GNPs alone or in a mixture with other chemotherapeutics. This can be translated into clinical applications for cancer patients.

### Supplementary Information

Supplementary Figure 1.

## Data Availability

All data are included in this published article.
