# Supplementary figures and images for: Combined oncolytic virotherapy gold nanoparticles as synergistic immunotherapy agent in breast cancer control
Source: Sci Rep. 2023 Oct 6;13:16843. doi: 10.1038/s41598-023-42299-4 (PMC10558528; doi:10.1038/s41598-023-42299-4)

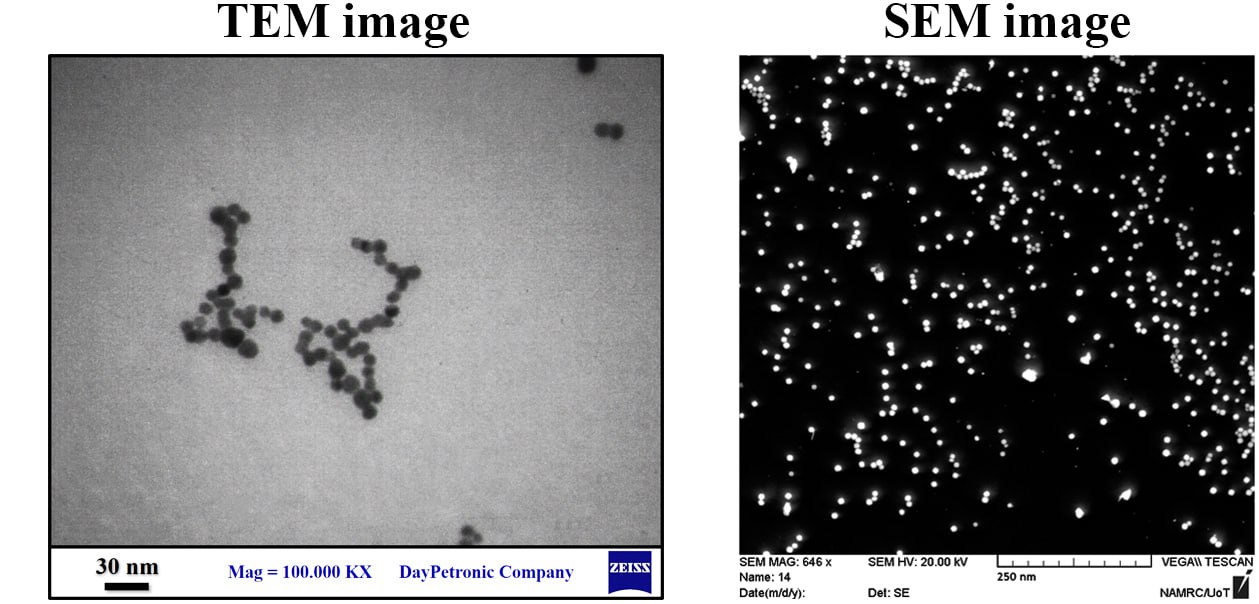


Supplementary Figure 1. TEM and SEM images of gold nanoparticles

Supplement: Supplementary file 1 — Supplementary Figure 1. [file 41598_2023_42299_MOESM1_ESM.docx]
